# Comparisons of Medical Student Knowledge Regarding Life-Threatening CT Images Before and After Clinical Experience

**Published:** 2017-08-30

**Authors:** Barbara Nguyen, Brady Werth, Nicholas Brewer, Jeanette G. Ward, R. Joseph Nold, James M. Haan

**Affiliations:** 1 University of Kansas School of Medicine-Wichita, Department of Surgery; 2 Via Christi Hospital Saint Francis, Wichita, KS; 3 Chandler Regional Medical Center, Chandler, Arizona

**Keywords:** x-ray computed tomography, medical students, knowledge, clinical clerkships

## Abstract

**Background:**

Currently, no national standard exists for educating medical students regarding radiography or formal research indicating the level of improvement regarding computed tomography (CT) interpretation of medical students during clinical rotations.

**Methods:**

Students were evaluated based on their response to twenty-two open-ended questions regarding diagnosis and treatment of eleven de-identified CT images of life-threatening injuries. The number of incorrect answers was compared with correct or partially correct answers between students starting third-year clinical rotations and those starting their fourth year.

**Results:**

Survey results were collected from 65 of 65 (100%) beginning third-year students and 9 of 60 (15%) beginning fourth-year students. Students in their fourth-year had less incorrect answers compared to third-year students, with five questions reflecting a statistically significant reduction in incorrect responses. The image with the least incorrect for both groups was epidural hemorrhage, 33.9% and 18.5% incorrect for third-year students for diagnosis and treatment, respectively, and 11.1% and 0% incorrect for fourth-year students. Outside of this image, the range of incorrect answers for third-year students was 75.4% to 100% and 44.4% to 100% for fourth-year students.

**Conclusion:**

Baseline CT knowledge of medical students, regardless of clinical experience, indicated a strong deficit, as more students were incorrect than correct for the majority of CT images.

## Introduction

Currently, there are no national standards for educating medical students regarding radiography interpretation in the trauma population. Radiology clinical rotations were required in only a quarter of U.S. medical schools as recently as 2009 to 2010.^1^ Among U.S. medical students surveyed, over three-fourths planned to take a radiology elective before residency.^2^ The majority of these students believed radiology changes patient care or was as important as a physical exam.^2^ In addition, general surgery program directors reported the ability to read abdominal x-rays and computed tomography (CT) imaging, among other radiologic-related tasks, as essential capabilities for incoming residents.^3^,^4^

Introduction to radiology in the clinical or hospital setting, even in the early phases of a student’s medical education, can influence their perception of imaging interpretation.^3^,^5^,^6^ However, institutions vary with regard to incorporation of radiology training. One approach that has shown success is the integration of medical imaging with an anatomy course.^6^–^10^ A study at Boston University School of Medicine evaluated the impact of CT scans of cadavers on students’ anatomy education and spatial relationships, with positive results.^6^ Two similar studies also found that inclusion of CT images in cadaver labs yields positive student perspectives and significant improvement in radiology skills.^9^,^10^

Although the literature provides numerous examples of studies related to medical student interpretation of radiographs, no formal study specifically has indicated the level of improvement of CT knowledge after one year of clinical rotation for U.S. medical students.^11^–^14^ Therefore, the purpose of this study was to observe and compare the baseline knowledge regarding CT interpretation of traumatic injuries for medical students starting clinical rotations to those completing their required rotations. Evaluating the extent of radiographic knowledge gained from clinical rotations alone in a setting lacking an emphasis on radiology education would provide the impacting factor. From this analysis, improvements and future discussion can be made regarding basic radiologic knowledge for medical students.

## Methods

All volunteer medical students tested were from the University of Kansas School of Medicine-Wichita (KUSM-W). The medical student curriculum at KUSM-W incorporates two years of didactic learning, followed by two years of clinical rotations. Radiology-specific education is not integrated into the core curriculum. However, during the surgical clinical rotation students partake in overnight trauma. Surgery residents are to involve medical students as appropriate; introduction to and education of CT imaging are expected.

Two separate groups were utilized for comparison: medical students beginning their third-year of clinical rotations (MS3), and medical students who recently had completed their third-year and were beginning their fourth-year of clinical rotations (MS4). Following informed consent, both groups participated in a timed, open-ended survey to evaluate their ability to interpret CT images typical of high-risk trauma situations.

De-identified single images of 11 different CT scans representing potential life-threatening injuries were identified by a board-certified trauma surgeon (Table 1). There were two questions per CT, for a total of 22 questions. Students were asked to identify: 1) the correct diagnosis, and 2) the correct treatment for the correct diagnosis. The students were instructed that each CT scan represented a life-threatening injury, as determined by two trauma surgeons, each having completed a fellowship in trauma and critical care surgery. Both groups were exposed to the same images in a controlled setting and were given a maximum of two minutes to view each image and record their interpretation of the image regarding diagnosis and treatment.

Survey forms were de-identified when scored and were reviewed separately and scored by two trauma surgeons. One point was assigned for a correct response, half a point for a partially correct response, or zero points for an incorrect response. Each student’s scores were averaged for each of the 22 questions. Both groups were compared by total points for each image, and proportion of incorrect responses was calculated by group. Differences in proportions were calculated.

Due to sample size, focus on the error reduction comparison was viewed through proportional differences, assessing statistical significance with a 95% confidence interval, corrected for continuity. Confidence intervals of 95% were calculated, of which those intervals, including zero, were considered statistically insignificant. Bonferroni correction indicated one out of 18 comparisons, given this confidence interval. Analyses were conducted using SPSS release 19.0 (IBM Corp, Somers, New York). The study was approved by the Institutional Review Board of Via Christi Hospitals, Wichita, Inc.

## Results

**Figure 1:**
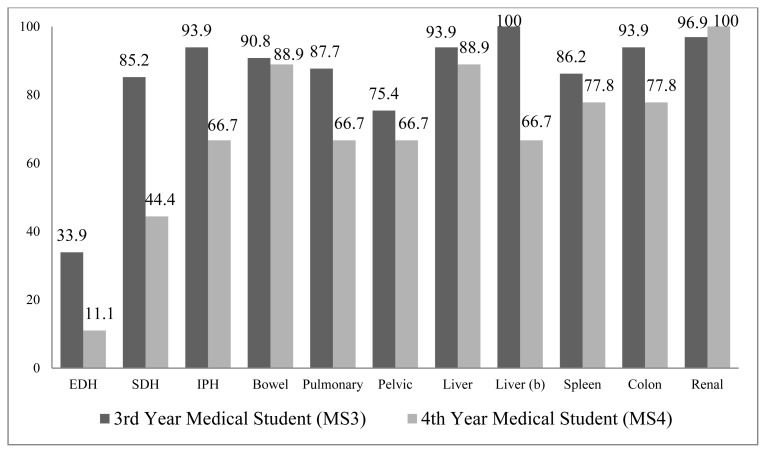
Percentage of incorrect responses for diagnosis by group.

**Figure 2:**
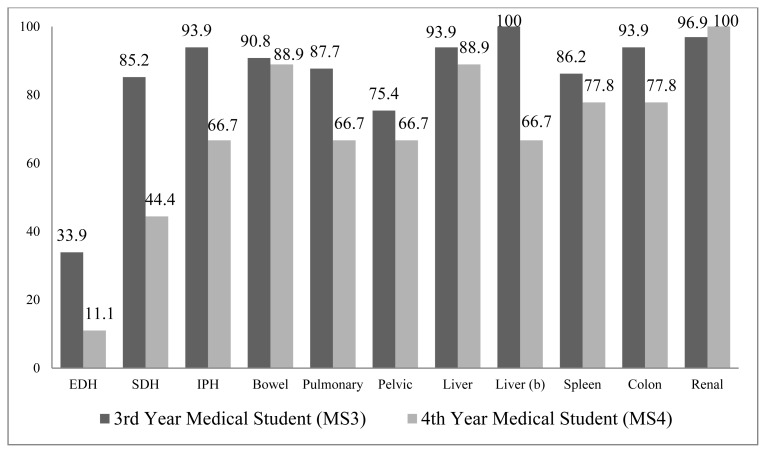
Percentage of incorrect responses by treatment by group.

Survey results were collected from 65 of 65 (100%) MS3s and 9 of 60 (15%) MS4s (N=74). Overall, MS4s performed better than MS3s, with fewer incorrect responses on 20 of 22 questions (Figures 1 and 2). Two exceptions were noted: diagnosis of a grade IV renal injury (96.9% incorrect for MS3s and 100% incorrect for MS4s) and treatment of a grade III liver laceration (95.4% incorrect for MS3s and 100% incorrect for MS4s). Neither were statistically significant (95% CI: 7.2 to 1.1% and −9.7 to 0.49%, respectively).

Percentage of incorrect responses ranges from 18.5% to 100% for MS3s and from 0.0% to 100% for MS4s. The image with the least incorrect responses by both groups was epidural hemorrhage, 33.9% and 18.5% incorrect by MS3s for diagnosis and treatment, respectively, and 11.1% and 0% for MS4s. Excluding epidural hemorrhage, the range of incorrect for MS3s was 75.4% to 100% and 44.4% to 100% for MS4s. The median percentage of incorrect responses for MS3s was 93.1% and 77.8% for MS4s. No MS3s were able to identify or propose a correct diagnoses or treatment for one of two grade III liver lacerations. No MS4s correctly diagnosed the grade IV renal injury and no MS4s correctly identified a treatment for one of two grade III liver lacerations.

Five of the twenty-two questions reflected a statistically significant reduction in incorrect responses between MS3s and MS4s. These included: diagnosis of subdural hemorrhage (85.2% incorrect MS3 versus 44.4% MS4, 95% CI: 8.2% to 75%); diagnosis of grade III liver laceration (100% MS3 versus 66.7% MS4, 95% CI: 2.5% to 64%); treatment of epidural hematoma (18.5% incorrect by MS3 versus all correct by MS4, 95% CI: 9.0% to 28%); treatment of small bowel thickening (98.5% MS3 versus 66.7% MS4, 95% CI: 0.85% to 63%); and treatment of right colon mesenteric injury (90.8% MS3 versus 55.6% MS4, 95% CI: 2.0% to 68%).

## Discussion

By comparing pre- and post-evaluations, several studies have demonstrated that medical students CT interpretation abilities improve with radiology-focused training.^15^–^17^ Sendra-Portero et al.^15^ and Scheiner et al.^16^ compared medical students of different years of study and assessed their abilities in interpreting radiology images before and after a radiology-specific training. Results indicated that medical students improved in interpreting radiographs after the training, regardless of year of study.^15^,^16^ Dawes et al.^17^ also found medical students to improve in interpreting radiographs after participating in a 26-week clinical training course. Results showed the proportion of correct answers improved from 8% pre-evaluation to 43% post-evaluation (p<0.001). Our study had similar results; however, the improvement was only slightly better for fourth-year students.

Of the twenty-two possible answers, MS4s did better than MS3s in all but two, indicating some improvement in reading CT imaging. However, both groups had a high range of incorrect responses. A median value of 93.1% incorrect by MS3s indicated the baseline CT knowledge of medical students entering clinical rotations was extremely low. As such, it is not surprising that MS4s had a mild improvement. Other than diagnosis and treatment of epidural hemorrhage, MS3s scored greater than 75% incorrect on all other questions. Although the median value for MS4s at 77.8% incorrect was lower than MS3s, it indicated a majority of students incorrectly identifying and ultimately treating injuries seen on CT scans.

Both groups performed best in diagnosing and treating an epidural hematoma. As MS3s did best with this injury, it raises the question if prior didactic learning regarding head injuries may have better prepared the students. However, MS3s did not perform particularly better on the other head injuries when compared to MS4s. No particular trend was noted regarding percentage incorrect and body regions.

Most surgeons expect MS3s to have a baseline ability to read CT scans and MS4s are expected to be able to identify life-threating images. Yet, this study showed a strong deficit in baseline CT knowledge amongst medical students. Despite the low number of MS4s, after a year of clinical rotations and overnight trauma calls, which included education on CT imaging, they did show some improvement in evaluating CT images. However, without a standard curriculum in radiology, it is difficult to conclude if this improvement is significant enough to warrant satisfactory expectations of a beginning fourth-year student. Still, for medical education purposes, clinical rotations appear to provide a benefit to students in education of CT imaging of life-threatening injuries.

Areas for potential study include improving or expanding upon the findings of the current study. Within the study, the benefits of viewing an entire CT scan may provide a more thorough investigation of student abilities. In addition, after completion of this study, a new radiology rotation was implemented. This new program may impact the current study results, therefore, a possible follow-up study may be beneficial.

Similar studies at other institutions may provide groundwork for average radiographic education, but without national standards or expectations, it would be difficult to conclude if an intervention is necessary. Future studies confirming these results might include comparison of students who had received imaging-specific education, building upon studies such as at Boston University School of Medicine.^6^ At the campus in this study, students are allowed to undergo a radiology elective in the fourth year. Comparisons between students who choose this elective with those who choose alternative electives may provide more solid results upon the benefits of imaging-specific education.

The most appropriate intervention from this study would be continued comparisons after clinical rotations with the addition of imaging-specific education. For our studies, pursuing a continued study of current MS3s at the end of their third-year would provide stronger conclusions on the effect of radiographic-specific education, as baseline CT knowledge already has been attained. Through this, more studies can be developed to achieve a thorough understanding of medical students’ radiographic knowledge, as well as how to improve their education and help them achieve optimal competency for residency and as physicians.

This study had several limitations. As this was a voluntary study with no compensation provided to participants, there were no expectations for subject participation numbers, although similar numbers were anticipated from both groups. However, due to the small number of fourth-year medical students, this group is not represented adequately. Further, the study tested students with one image slice of a CT scan as opposed to a complete CT scan set, a scenario unlikely in real life. Comparative slices on a CT scan may help the reader better analyze the severity and extent of the injury. Also, the patient’s clinical history could have assisted students in their evaluations. Finally, the findings may not be generalizable to other trauma centers since this study was conducted at a single center.

## Conclusions

The high percentage of incorrect responses reflects a strong deficit in baseline CT knowledge amongst medical students, particularly in an environment with limited radiologic education. Outside of diagnosis and treatment of head injuries, most students from both groups answered diagnosis and treatment incorrectly for the majority of the scans. If more than half of students are expected to identify these images correctly, other interventions are necessary to ensure better radiographic education. This study argues that a re-evaluation of current standards for radiographic education of medical students is needed. In addition, the possibilities of how to implement this education should be considered, whether it is through utilization of radiologist involvement and/or curriculum-specific education.

## Figures and Tables

**Table 1:** Life-threatening computed tomography injury and treatment images.

Injury Type	Treatment
Head	
Epidural hematoma (EDH)	Operative intervention or drainage
Subdural hematoma (SDH)	Medical management
Intraparenchymal hemorrhage (IPH)	Medical management
Chest, Abdomen and Pelvis	
Pulmonary contusion and pneumothorax	Chest tube
Grade III liver laceration	Observation in the intensive care unit
Grade III liver laceration	Observation in the intensive care unit
Grade IV splenic injury	Embolization or operative intervention
Grade IV renal injury	Embolization or operative intervention
Pelvic fracture	Operative intervention or sacroiliac screw
Small bowel thickening	Serial exam or operative intervention
Right colon mesenteric injury	Serial exam or operative intervention
